# A clinical evaluation of an *ex vivo* organ culture system to predict patient response to cancer therapy

**DOI:** 10.3389/fmed.2023.1221484

**Published:** 2023-09-28

**Authors:** Shay Golan, Vered Bar, Seth J. Salpeter, Guy Neev, German Creiderman, Daniel Kedar, Sara Aharon, Lubov Turovsky, Adi Zundelevich, Hamutal Shahar, Hagit Shapira, Giuseppe Mallel, Erez Stossel, Nancy Gavert, Ravid Straussman, Zohar Dotan, Raanan Berger, Chani Stossel, Talia Golan, Sharon Halperin, Dan Leibovici, Shani Breuer, Yakir Rottenberg, Liat Applebaum, Ayala Hubert, Hovav Nechushtan, Tamar Peretz, Aviad Zick, Boris Chertin, Dmitry Koulikov, Amir Sonnenblick, Eli Rosenbaum

**Affiliations:** ^1^Department of Urology, Beilinson Hospital – Rabin Medical Center, Petah Tikva, Israel; ^2^Curesponse, Rehovot, Israel; ^3^Department of Molecular Cell Biology, The Weizmann Institute of Science, Rehovot, Israel; ^4^Department of Oncology, Sheba Medical Center, Ramat Gan, Israel; ^5^Department of Urology, Kaplan Medical Center, Rehovot, Israel; ^6^Sharett Institute of Oncology, Hadassah Medical Organization and Faculty of Medicine, Hebrew University of Jerusalem, Jerusalem, Israel; ^7^Department of Urology, Shaare Zedek Medical Center, Jerusalem, Israel; ^8^Department of Oncology, Tel Aviv Sourasky Medical Center, Tel Aviv, Israel; ^9^Sackler Faculty of Medicine, Tel Aviv University, Tel Aviv, Israel; ^10^Institute of Oncology, Davidoff Cancer Center, Rabin Medical Center, Petah Tikva, Israel

**Keywords:** cancer, solid tumors, *ex vivo* organ culture, clinical response, prediction of treatment response

## Abstract

**Introduction:**

*Ex vivo* organ cultures (EVOC) were recently optimized to sustain cancer tissue for 5 days with its complete microenvironment. We examined the ability of an EVOC platform to predict patient response to cancer therapy.

**Methods:**

A multicenter, prospective, single-arm observational trial. Samples were obtained from patients with newly diagnosed bladder cancer who underwent transurethral resection of bladder tumor and from core needle biopsies of patients with metastatic cancer. The tumors were cut into 250 μM slices and cultured within 24 h, then incubated for 96 h with vehicle or intended to treat drug. The cultures were then fixed and stained to analyze their morphology and cell viability. Each EVOC was given a score based on cell viability, level of damage, and Ki67 proliferation, and the scores were correlated with the patients’ clinical response assessed by pathology or Response Evaluation Criteria in Solid Tumors (RECIST).

**Results:**

The cancer tissue and microenvironment, including endothelial and immune cells, were preserved at high viability with continued cell division for 5 days, demonstrating active cell signaling dynamics. A total of 34 cancer samples were tested by the platform and were correlated with clinical results. A higher EVOC score was correlated with better clinical response. The EVOC system showed a predictive specificity of 77.7% (7/9, 95% CI 0.4–0.97) and a sensitivity of 96% (24/25, 95% CI 0.80–0.99).

**Conclusion:**

EVOC cultured for 5 days showed high sensitivity and specificity for predicting clinical response to therapy among patients with muscle-invasive bladder cancer and other solid tumors.

## Introduction

Despite the widespread acceptance of genomic sequencing as an integral part of cancer personalized medicine, highly accurate individual patient drug selection remains a major unsolved problem. Targeted genetic diagnostics are used as high-fidelity companion biomarkers for inhibitors of molecular pathways, yet broad-based genomic sequencing aimed at drug selection has proven inadequate for improving patient response and outcome (1, 2). For example, the SHIVA study, a randomized trial of genomic-based precision medicine, did not show a benefit in progression-free survival for patients assigned to genome-based treatment when compared to physicians’ choice (3, 4).

Genomic-based drug selection may improve patient outcomes when combined with a functional platform capable of assessing the effect of specific drugs on a patient’s tumor sample. Initially, several approaches evaluated the predictive capacity of functional assays by dissociating the tumor and testing drugs on patient cancer cells (5, 6) or by growing patient cancer avatars in patient-derived xenograft (PDX) mouse models. More recently several groups have examined the potential of organoids as a functional assay for modeling patient drug response (1, 7). These systems do not account for the complex role of the tumor microenvironment in regulating the response to therapy. Multiple stromal components such as immune cells, fibroblasts, blood vessels, and even bacteria have been shown to affect tumor response to treatment, suggesting the need for their inclusion in functional predictive assays (8–10).

While *ex vivo* organ culture (EVOC) has been utilized for decades, it was only recently optimized to sustain cancer tissue for 5 days with its complete microenvironment (11–14). Although EVOC platforms are not amenable to high throughput screening due to the limited amount of tumor tissue, they can help to assess potential drug responses to several drugs and combinations a patient may receive. Moreover, as opposed to organoids and PDX models that require weeks and months to establish, EVOC provides rapid results and therefore can potentially be used for immediate (within days) treatment decision-making. Recently, a new EVOC platform was developed showing exceptional ability to preserve a broad variety of cancer types with the surrounding microenvironment for an extended period (15). This approach, which enables culturing thick sections of several different cancer types for 5 days, was shown to accurately predict the drug response of numerous tumor types compared to the *in vivo* PDX response and showed correlation with predictive genomic biomarkers of human tumors (15).

We have conducted a multicenter, prospective, single-arm observational trial to examine the ability of this EVOC platform to predict patient response to therapy in the clinic. First, we have recapitulated the previously shown capacity of the system for preserving resected patient cancer tissue with its microenvironment over several days. Next, we established a predictive clinical trial in muscle-invasive bladder cancer (MIBC) to evaluate the accuracy of the platform’s prediction of drug response in patients receiving neoadjuvant or induction chemotherapy where up to 30% of patients are not expected to respond to therapy (15). MIBC was chosen as a first indication, since significant quantities of resected tissue are frequently available for EVOC profiling, and the patient’s response can be followed throughout their treatment. To further validate the capacity of the platform to preserve tissue and predict patient response based on core biopsy samples, we assembled a cohort of patients with different metastatic cancers who underwent biopsies prior to treatment. Finally, the platform’s results were compared to the patients’ clinical response using pathology scores or Response Evaluation Criteria in Solid Tumors (RECIST).

## Patients and methods

### Bladder cancer

To determine the predictive capacity of the EVOC system, we first established a clinical trial in patients with bladder cancer who were candidates for neoadjuvant or induction chemotherapy. Patients with newly diagnosed bladder cancer, who were referred for transurethral resection of bladder tumor (TURBT) at four medical centers, were considered eligible for this study. All patients underwent axial imaging [either computed tomography (CT) or positron-emission tomography (PET)/CT] for staging at diagnosis. At the beginning of the TURBT, a 1–3 mL sample was obtained from the tumor’s most superficial area and far from its base to avoid any impact on local histological staging. The sample was immediately placed in ice-cold Dulbecco’s Modified Eagle’s Medium (DMEM) and transferred to the lab. The final referral to chemotherapy was at the urologist’s and oncologist’s discretion.

### Core biopsies of metastatic tumors

Patients with highly suspected metastatic cancer, or those previously diagnosed with metastatic cancer, who underwent axial imaging (either CT or PET/CT) for staging of pancreatic, breast, liver, colon, sarcoma, or esophageal tumors (at least 2 cm in diameter), and referred for diagnostic needle core biopsy at three medical centers, were eligible for participating in the study. During the procedure 2–4 cores of 16 gauge or 18 gauge needles were removed and transferred to the lab in ice-cold DMEM.

### Preparation of the EVOC

As previously described (11), tumors were cut into 250 μM slices using a vibratome (VF300, Precisionary Instruments). A sample of the tissue was fixed immediately in 4% paraformaldehyde (PFA) as a reference and analyzed for viability within 24 h. The rest of the slices were placed in 12 or 24 well plates on titanium grids with 4 mL of DMEM/F12 medium [supplemented with 5% fetal calf serum (FCS), penicillin 100 IU/mL with streptomycin 100 μg/mL, amphotericin B 2.5 μg/mL, gentamicin sulfate 50 mg/mL, and L-glutamine 100 μL/mL]. The tissue slices were then cultured at 70 rpm on an orbital shaker (TOU-120 N, MRC) at 37°C, 5% CO_2_, and 80% O_2_. One day after sectioning, bladder tumor sections were treated with cisplatin 30 μM and/or gemcitabine 30 μM for 96 h, with media and drug change after 48 h.

Core biopsies obtained from patients with metastatic cancer study (2–5 core biopsies/patient) were sectioned as described above. One tissue section was fixed immediately as a reference and assessed by rapid histology within 24 h. The remaining tissue was prepared as EVOC and cultured with drugs that were likely to be used in the upcoming clinical treatment as suggested by the treating oncologist. Drug concentrations are listed in Supplementary Table S1 including concentrations that were described previously (11, 12). Unless otherwise noted, drugs were cultured for 96 h and medium change after 48 h. At the end of the incubation period the tissue sections were fixed overnight with 4% PFA followed by formalin-fixed paraffin embedding (FFPE).

To monitor pathway regulation in response to drug treatment, tissue samples were cultured with small molecule inhibitors of several oncogenic pathways for 24 h: trametinib 10 nM (a pERK inhibitor), palbociclib 10 μM (a CDK4/6 inhibitor) and NT219 20 μM [a pStat3/insulin receptor substrate (IRS) inhibitor]. The samples were then stained by immunohistochemistry.

### EVOC scoring

Tissue immunohistochemistry was performed on 4 μm sections from the FFPE tissue samples. Hematoxylin and eosin (H&E) staining was performed using an automated stainer (Leica Biosystems). Ki67 staining (Thermo Fischer Antibody (RM-9106); 1:500 dilution) was performed using an automated stainer (BOND RX, Leica Biosystems). The pathologists were shown the tissue fixed immediately after it was resected from the patient and sliced (time 0) and an untreated tissue obtained after 5 days (control) as reference samples. All other treated EVOC samples were evaluated blindly. The pathologists assessed the viability (*Vi*) of live treated tumor cells on a scale of 0–100% (compared to the immediately fixed and control samples), the level of damage (*Q*) on a scale of 0–4, and Ki67 proliferation (*K*) factored as a percentage of replicating cells. To account for tissue heterogeneity, the scores are an average of the treated tissue with a particular drug from 3 different tumor slices taken from the first third, middle third and final third of the biopsied specimen. A final score on a scale of 0–100, accounting for all parameters, was obtained using the formula:


$$\overset{c}{\underset{a}{\sum }}\Delta Vi_{T_{0}}^{V^{100}}*X+QT_{0}^{4}*Y+K_{0}^{100}*Z$$

A score of 0 represents completely viable cancer cells, suggesting no response, and a score of 100 represents no viable cancer cells, suggesting complete response. The weighted output of the evaluation was based on coefficients *X*, *Y*, *Z* corresponding to 0.7, 0.2, and 0.1, respectively. A threshold score of 45 was used to differentiate between non-responders and responders based on the complete training set applied using the above coefficients on MIBC bladder samples correlated to historical clinical response for neoadjuvant treatment.

### Standard of clinical reference

In patients with MIBC treated with neoadjuvant gemcitabine and cisplatin, followed by radical cystectomy (RC), the pathological evaluation of the surgical specimen served as the standard of reference. Response to treatment by pathology was defined as 1. complete response (no malignancy present – pT0), 2. partial response (histological downstaging), or 3. no response (no change of histological staging or upstaging). In patients with bladder cancer who received chemotherapy without undergoing surgery, radiological follow-up data was used according to the RECIST criteria (16). In the core biopsy cohort, RECIST 1.1 criteria were based on imaging.

### Genomic sequencing

Patients from the metastatic biopsy study underwent genomic sequencing on gDNA extracted from the tumor sample using the Qiagen Dneasy DNA purification kit. The test is based on next generation sequencing (NGS) using iSeq100 of a panel containing 286 amplicons covering hotspots of 57 genes relevant to cancer and treatment (Swift Biosciences, Inc.). Genomic analysis for mutation frequencies greater than 5% is presented.

### Setting and patients

The study was conducted at 6 medical centers across Israel and its protocol was approved by the institutional ethics committees (refer to Supplementary Table S2 for medical center names and ethics committee approval numbers). All patients provided their informed consent prior to enrolling in the study.

### Statistical analysis

Data were analyzed by descriptive statistics. Sensitivity, specificity, positive predictive value (PPV) and negative predictive value (NPV) of the EVOC tumor response scores were calculated. Differences in tissue viability, Ki67 staining, or EVOC scores and correlations to different clinical outcomes were evaluated using *t*-test or analysis of variance. *p* values <0.05 were considered statistically significant. All statistical analyses were conducted using SPSS 20 (IBM Corp.).

## Results

### The EVOC system preserves the tumor microenvironment

**Figure 1:**
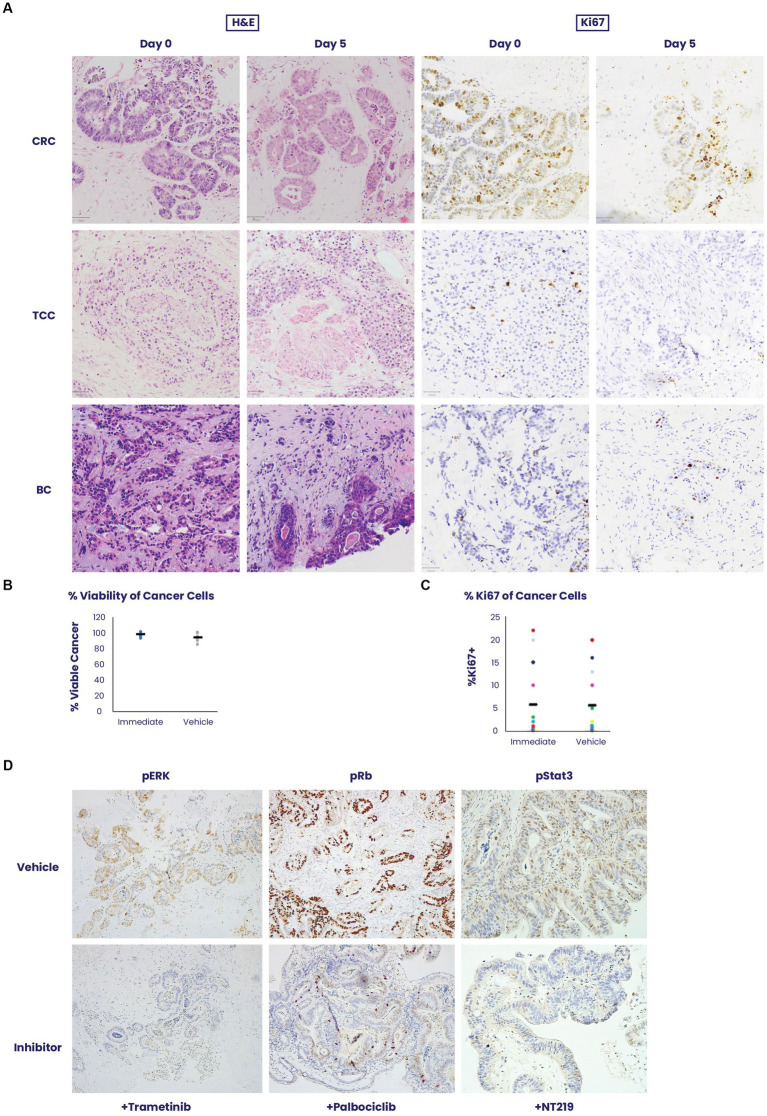
An *ex vivo* organ culture (EVOC) system for preservation of the cancer microenvironment. **(A)** Colorectal cancer (CRC), transitional cell carcinoma (TCC) and breast cancer (BC) were cultured in an EVOC assay that preserves the tumor microenvironment. Representative images of the tissues are shown on Day 0 – when the tissue was received and after 5 days in culture. Hematoxylin and eosin (H&E) and Ki67 staining are shown. **(B,C)** Viability and proliferation of the different cancer samples were compared between Day 0 and Day 5 (*N* = 5 per cancer type). Cancer viability and Ki67% stain was assessed and quantified by a pathologist showing non-significant changes during the culture period (*N* = 16). **(D)** The capacity for signal transduction modification was assessed by adding specific pathway inhibitors and determining their effect after 24 h. Trametinib, a pERK inhibitor, palbociclib, a CDK4/6 inhibitor and NT219, a pStat3/insulin receptor substrate (IRS) inhibitor were added to the culture and their respective pathway targets were stained by immunohistochemistry showing downregulation of activity in the culture system.

To validate the robustness of the EVOC technology, which allows to maintain thick sections of different cancer types in culture for 5 days (11), we examined the survival and cell dynamics of colorectal cancer, urothelial carcinoma and breast cancer (Figure 1A). All tissue types showed a mean 95% viability on day 5 when compared to the tissue sampled immediately after surgical removal on day 0 (Figure 1B). Additionally, no statistically significant difference was noted in the average percentage of Ki67-positive cancer cells on day 0 (5.8%) versus day 5 (5.5%), *p* = 0.92, among samples of all tissue types (*n* = 16) of breast cancer, urothelial carcinoma and colorectal cancer (Figure 1C).

To assess signaling pathway dynamics in the tissue, the EVOC was cultured with small molecule inhibitors of several oncogenic pathways for 24 h and evaluated for the ability to suppress signaling using phosphorylated markers of signaling dynamics. Corresponding to their respective pathways, trametinib downregulated pERK, palbociclib lowered pRB and NT219 blocked pStat3 staining (Figure 1D). These results illustrate the sensitivity of the tissue to specific drug inhibition and the corresponding response shown by the signaling pathway.

### EVOC of samples from patients with MIBC

In total, 111 patients with newly diagnosed bladder tumors yielded 101 samples that were processed in the EVOC system (Supplementary Figure S1). Fifty-one of these samples were non-muscle-invasive urothelial carcinomas (Ta/T1), and 50 were muscle-invasive urothelial cancers (T2). The clinical characteristics of all patients are shown in Supplementary Table S3.

**Figure 2:**
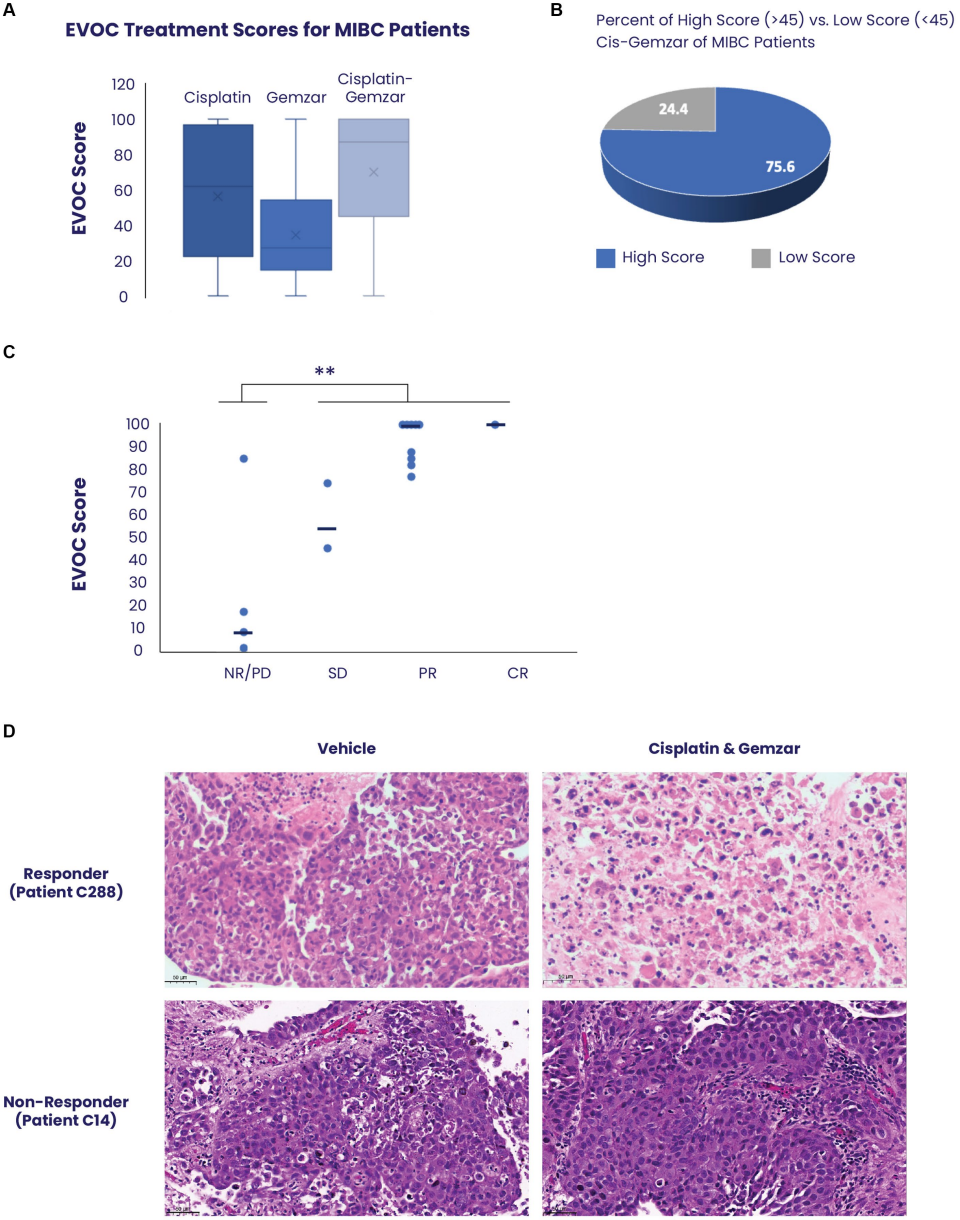
Correlation between EVOC scores and clinical response of patients with MIBC. **(A)** EVOC scores were computed for each sample treated with cisplatin alone, gemcitabine alone and combined cisplatin and gemcitabine. The average is marked with an X while median is denoted by a horizontal line **(B)** Using an EVOC score of 45 to differentiate between non-response and response we found that 75.6% of patients with MIBC who had an EVOC score were classified as responders while 24.4% were classified as non-responders. **(C)** The clinical outcomes (pathology or RECIST) of patients who received a full course of cisplatin and gemcitabine were correlated to their EVOC score. EVOC scores are listed next to the four patients who were found to be non-responders (red) and the twelve patients identified as responders (blue). Patients who underwent cystectomy were listed as non-responders (NR), partial responder (PR), and complete responder (CR) based on pathology, while tumors of patients who were evaluated by imaging alone were designated a RECIST score [progressive disease (PD), stable disease (SD), PR, or CR]. **(D)** Representative H&E images from a responder and non-responder patient with bladder cancer. Vehicle samples were compared to samples treated with cisplatin and gemcitabine for 5 days.

MIBC EVOC scores were obtained from 46 samples (Figure 2A). Samples treated with cisplatin showed a median score of 64 (mean 59) while those treated with gemcitabine scored a median of 27 (mean 34), indicating a significantly greater effect of cisplatin therapy. Combined treatment with cisplatin and gemcitabine showed a median score of 87 (mean 70), indicating a combinatorial effect of the treatments. Notably, analysis of all cisplatin-gemcitabine scores showed high response scores (≥45) in 75.6% (35/46) of samples compared to low scores (<45) in 24.4% (9/46) corresponding to values found in the literature for response to neoadjuvant bladder cancer therapy (Figure 2B).

### EVOC score predicts clinical response to therapy in MIBC

Among the 50 patients with MIBC, 16 (32%) received a full course of chemotherapy with cisplatin and gemcitabine, and their clinical response was correlated with the EVOC scores. Based on the previously noted distinction between the high and low EVOC scores, an EVOC score of 45 was applied to differentiate between responders and non-responders. The clinical outcomes (pathology or RECIST) of patients who received a full course of cisplatin and gemcitabine were correlated to their EVOC score. Among the 16 patients who completed therapy, 12 were responders while 4 were non-responders based on final pathology or RECIST (Table 1). A correlation was observed between clinical response and EVOC scores [*p* = 0.04 by *t* test comparing progressive disease (PD)/no response (NR) scores to stable disease (SD)/partial response (PR)/complete response (CR) scores]. Higher median EVOC scores were correlated with better clinical response category: a median EVOC score of 8 for PD (mean 27.5), 59.5 for SD (mean 59.5), 100 for PR (mean 92.4), and 100 for CR (mean 100) (Figure 2C).

**Table 1:** EVOC score and clinical response of bladder cancer patients who completed therapy.

Count	Patient	Tissue type	Treatment	EVOC score	Clinical response
1	C51	Bladder	Cisplatin-gemcitabine	0	NR (Pathology)
2	C14	Bladder	Cisplatin-gemcitabine	8	NR (Pathology)
3	C35	Bladder	Cisplatin-gemcitabine	17	PD (RECIST)
4	C139	Bladder	Cisplatin-gemcitabine	45	SD (RECIST)
5	C130	Bladder	Cisplatin-gemcitabine	74	SD (RECIST)
6	C105	Bladder	Cisplatin-gemcitabine	77	PR (RECIST)
7	C63	Bladder	Cisplatin-gemcitabine	82	PR (Pathology)
8	C273	Bladder	Cisplatin-gemcitabine	85	PR (Pathology)
9	C288	Bladder	Cisplatin-gemcitabine	85	PD (RECIST)
10	C95	Bladder	Cisplatin-gemcitabine	88	PR (Pathology)
13	C21	Bladder	Cisplatin-gemcitabine	100	PR (Pathology)
14	C128	Bladder	Cisplatin-gemcitabine	100	PR (RECIST)
15	C190	Bladder	Cisplatin-gemcitabine	100	PR (RECIST)
16	C143	Bladder	Cisplatin-gemcitabine	100	PR (Pathology)

Black denotes responders and red denotes non-responders. EVOC, *ex vivo* organ culture; NR, no response; PD, progressive disease; PR, partial response; SD, stable disease; RECIST, response evaluation criteria in solid tumors.

Representative images of tissue response show significant cell death of urothelial carcinoma cells following treatment with cisplatin and gemcitabine, compared to control, in patients with clinically confirmed response. In contrast, tissue samples obtained from clinically confirmed non-responders show that urothelial carcinomas cells remain highly viable and refractory to treatment (Figure 2D; Supplementary Figure S2).

### EVOC scores in metastatic tumor specimens obtained by needle core biopsy

To further validate the application of the EVOC platform for predicting patient response, we established an additional cohort of patients with solid tumors, who underwent core needle biopsy samples known to be more challenging to maintain *ex vivo*. EVOC from core-needle biopsies of patients highly suspected of metastatic cancer or those previously diagnosed with metastatic cancer were compared to the clinical response of these patients.

**Figure 3:**
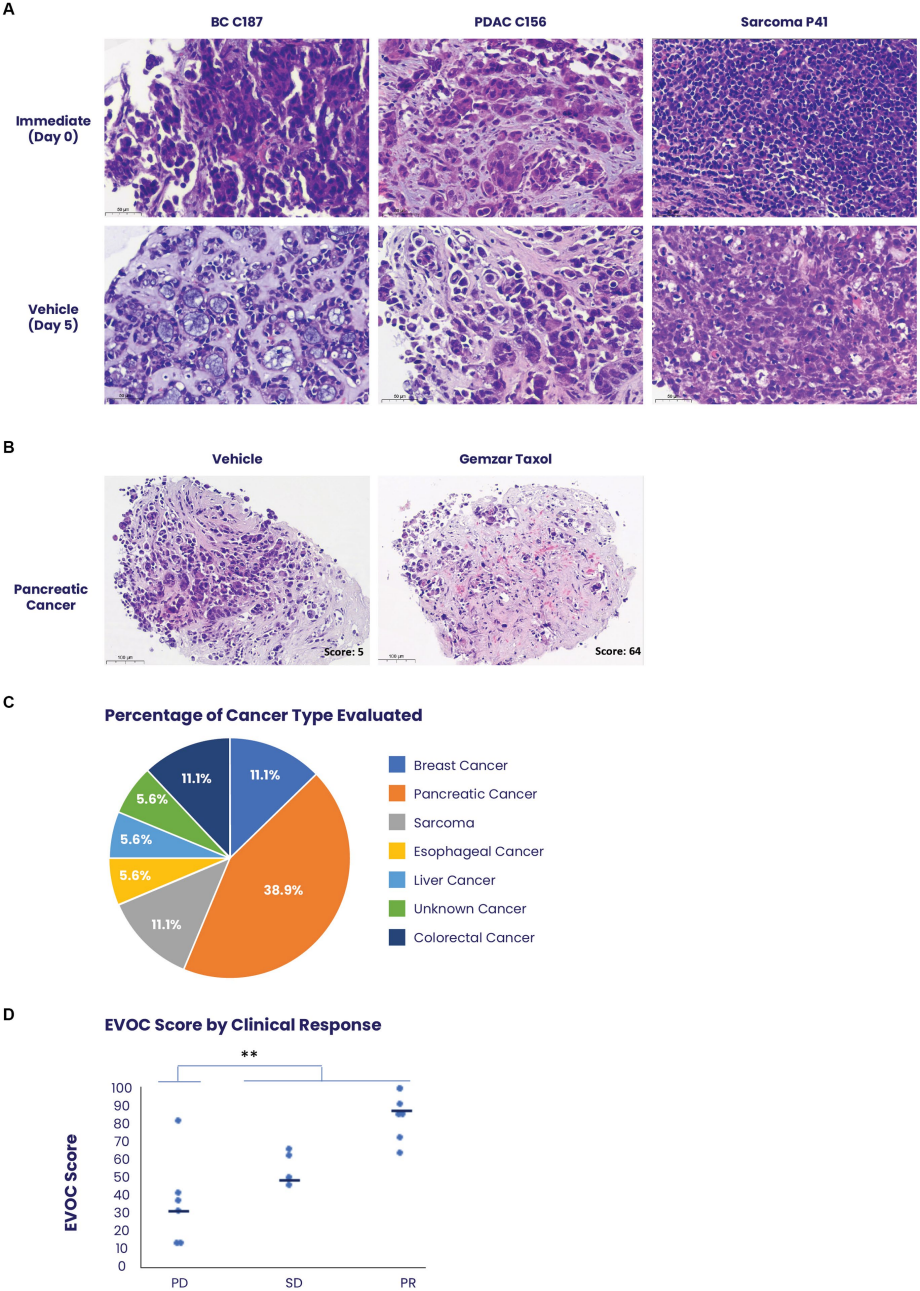
Use of the EVOC system to assess treatment response on biopsies. **(A)** Biopsies of breast cancer (BC), pancreatic ductal adenocarcinoma (PDAC) and scarcoma were obtained prior to initiation of patient treatment and maintained in EVOC for 5 days. Representative images of the biopsies on day 0 and day 5 show high viability and preservation during the culture assay of several prominent tissue types. **(B)** Representative images of a pancreatic cancer biopsy stained with hematoxylin & eosin comparing treatment with vehicle and treatment with combined paclitaxel and gemcitabine after 5 days. The sample treated with paclitaxel and gemcitabine shows cell-death representing a response and an EVOC score of 62. **(C)** A graph demonstrating the percent of each cancer type obtained in the clinical trial evaluating EVOC predictive capabilities on biopsies. **(D)** Correlation between EVOC scores and clinical outcomes of patients. Cancer types included pancreatic, breast, colorectal, esophageal, sarcoma, and liver cancer *N* = 18.

In total, 94 patients with metastatic tumors were recruited to the study. Fifty of these patients had at least 90% viable cancer tissue in the immediate sample taken from their core biopsy. Forty samples completed the process with at least 80% cancer cell viability in the vehicle sample, which was determined as the cut-off for a successful assay (Supplementary Figure S3). Representative images of biopsy samples of breast cancer, pancreatic ductal adenocarcinoma, and sarcoma, maintain tissue architecture, and viability after 5 days in culture (Figure 3A). Biopsy samples clinically identified as responders showed significant tissue death which corresponded to higher scores in the EVOC platform (Figure 3B).

Among the 40 patients with EVOC results, a total of 18 completed their prescribed course of treatment and had follow-up clinical data for correlation. The largest group of samples was pancreatic cancer (38.9%). Additional samples were breast, colorectal, esophageal, sarcoma, and liver cancers (Figure 3C). Genomic analysis was performed to identify notable genomic mutations (Supplementary Table S4) and demonstrates mutational heterogeneity among the tumor types. EVOC scores were then correlated with their corresponding clinical outcome (Table 2). Plotting the EVOC scores of each of the patients in their respective clinical response categories showed a significant difference between the scores of responders and non-responders (*t*-test *p* < 0.001 comparing PD/NR scores to SD/PR/CR scores). Moreover, higher median EVOC scores were correlated with better clinical response categories: a median EVOC score of 31 for PD (mean 22.6), 50 for SD (mean 56), and 83.1 for PR (mean 83.1) (Figure 3D).

**Table 2:** EVOC score and clinical response of patients with other cancer types who completed therapy.

Patient	Tissue type	Treatment	EVOC score	Clinical response
P55	Sarcoma	Pazopanib-everolimus	13	PD
C229	Esophageal	Paclitaxel	13	PD
C150	Colorectal cancer	FOLFOX-cetuximab	31	PR
E93	Pancreas	FOLFIRNOX	37	PD
C243	Pancreas	Gemcitabine	41	PD
C159	Sarcoma	Ifosamide-etoposide	46	SD
C161	Pancreas	FOLFIRNOX	50	SD
C117	Pancreas	FOLFIRNOX	62	SD
C156	Pancreas	Gemcitabine-paclitaxel	64	PR
C107	Pancreas	FOLFIRNOX	66	SD
C168	Pancreas	FOLFIRNOX	72	PR
C170	Unknown	Cisplatin-gemcitabine	82	PD
C301	Breast	Alpelisib-letrozole	85	PR
C303	Breast	Eribulin	85	PR
C149	Colorectal cancer	FOLFOX	86	PR
C115	Pancreas	FOLFIRNOX	91	PR
C188	Unknown	Carboplatin-paclitaxel	100	PR
C261	Liver	FOLFIRNOX	100	PR

Black denotes responders and red denotes non-responders. EVOC, *ex vivo* organ culture; FOLFOX, leucovorin calcium (folinic acid), fluorouracil and oxaliplatin; FOLFIRINOX, leucovorin calcium (folinic acid), fluorouracil, irinotecan hydrochloride, and oxaliplatin; PD, progressive disease; PR, partial response; SD, stable disease.

### Correlation of EVOC scores with clinical response of patients for clinical prediction

**Figure 4:**
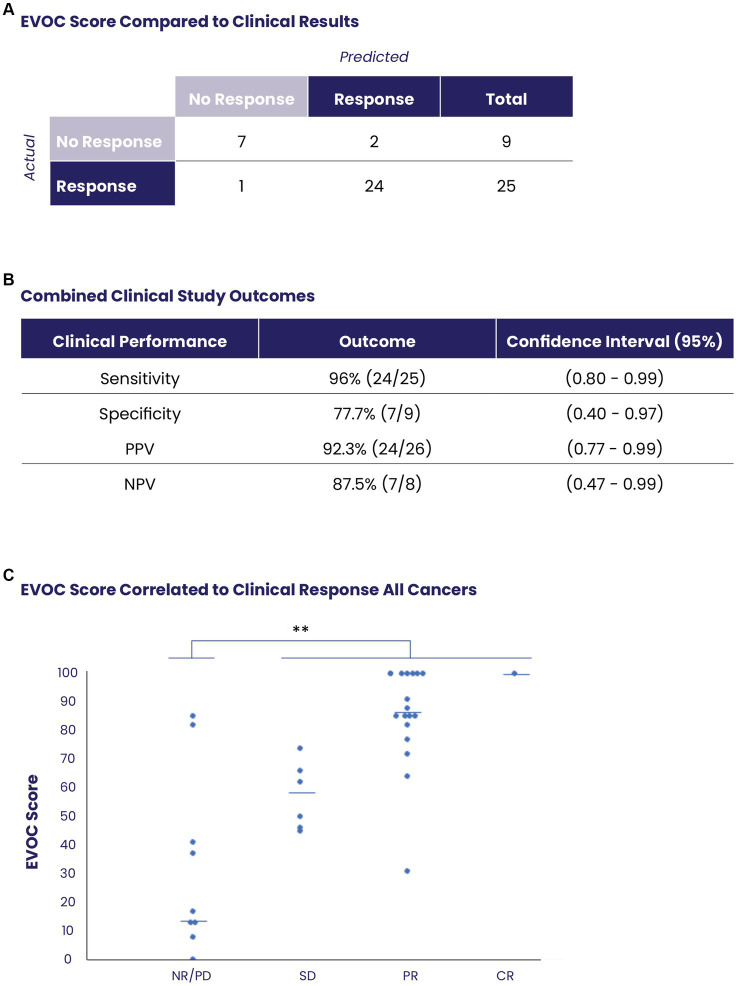
Prediction of clinical response using EVOC scores of all collected samples. **(A)** The distribution of responders and non-responders based on an EVOC score of 45. **(B)** Computation of predictive statistical calculations for sensitivity, specificity, positive predictive value (PPV) and negative predictive value (NPV). **(C)** Correlation between EVOC scores and clinical outcomes of patients (determined by pathology or RECIST) with resected or biopsy samples (*N* = 34).

Overall, EVOC results were correlated with clinical data in 34 patients (16 MIBC samples from TURBT and 18 other cancers from core needle biopsy). A threshold EVOC score of 45 provided an optimal distinction between responders and non-responders, correlating closely with clinical results (Figure 4A). Nine patients (26.4%) were non-responders to therapy (defined as PD in RECIST or NR on pathology) while 25 patients (73.5%) showed a response (SD, PR, CR in RECIST or PR, CR in pathology). The EVOC system showed a predictive specificity of 77.7% (7/9, 95% CI 0.4–0.97), a sensitivity of 96% (24/25, 95% CI 0.80–0.99), a PPV of 92.3% (24/26, 95% CI 0.77–0.99) and NPV of 87.5% (7/8, 95% CI 0.47–0.99) (Figure 4B). Comparison of EVOC scores of clinical responders with non-responders showed a statistically significant difference (*p* < 0.01). Moreover, comparison of scores from each clinical response category yielded median EVOC scores of 17 for PD (mean 32.88), 56 for SD (mean 57.16), 86 for PR (mean 84), and 100 for CR (mean 100), indicating that higher EVOC scores predict better clinical response (ANOVA *p* < 0.01, *F* = 15.1453) (Figure 4C).

## Discussion

Recent efforts to establish EVOC systems have yielded new approaches for cancer personalized medicine which are able to reproduce the tumor microenvironment (TME) and account for its effect on drug response for predictive cancer medicine (1, 11). Early attempts using cancer cell lines and monolayers devoid of stroma or immune tissue did not sufficiently predict patient response, and while organoids and organ-on-chip tissue cultures maintain some supporting cells they do not accurately recapitulate the TME as it is found in the patient (17–19).

In this study, we used an EVOC platform to predict patient treatment outcomes on both resected samples and core biopsies from other solid metastatic tumors. We showed that the EVOC platform preserves the viability of the cancer microenvironment of several cancer types for over 5 days. Using a threshold EVOC score of 45 to differentiate between responders (≥45) and non-responders (<45), we found an overall sensitivity of 96% and a specificity of 78% with higher scores corresponding to better clinical response.

Predictive precision cancer information must be delivered to the patient in a timely fashion so that it can be translated into actionable treatment decisions (14, 20). Even the most rapidly established patient organoid models require over a month to achieve sufficient culture material for drug assessment. PDX models require 5–6 months to establish sufficient colonies for drug profiling (7, 21). Current commercial genomic profiling is usually returned within 2–4 weeks, establishing a common turn-around time for information regarding treatment selection. The EVOC platform described here requires 6 days from the time the tissue arrives for fixation of sections. The blocks are then sectioned, stained, and evaluated within 4 days. This protocol could provide results to oncologists and their patients in approximately 2 weeks, which is comparable to the current standard of care for personalized cancer medicine.

Tissue volume and quality are important factors for achieving sustainable three-dimensional EVOC. In the previous paper reporting the use of this EVOC assay, only resected tissue was evaluated, and showed a high level of survival over 5–7 days in culture: 77.6% (114/147) of tissues received had viable cancer, and of those 91.2% (104/114) of resected tissues were viable in the EVOC and produced a score (11). Consistent with these data, despite the use of cautery during TURBT (for hemostatic purposes), 95% of resected MIBC samples had viable cancer tissue (101/106) and 92% (46/50) produced viable EVOC scores. These findings strongly support the future applicability of this system in patients with bladder cancer and other indications where resected tissue is available. Although the amount of tissue obtained by needle core biopsy is small, we showed that the tissue can be maintained in EVOC and that response to therapy can be evaluated in 80% of cases (40/50).

Many samples arrived without cancer tissue or with completely necrotic cancer tissue (46.8% of samples, 44/98) – a common problem in samples derived by core needle biopsy. Furthermore, as the samples for EVOC study were taken along with the ones required clinically, this problem was potentially exacerbated. To minimize this problem, in future studies patients should provide biopsies exclusively for EVOC. Additionally, obtaining biopsies by 16-gauge needles, will enable extraction of more tissue with higher quality, as shown in previous genomic studies (22, 23).

The ability to accurately predict patient response prior to treatment holds significant promise for improving patient outcomes and survival. Notably, analysis of all MIBC EVOC cultures treated with cisplatin and gemcitabine showed high response scores (≥45) in 75.6% (35/46) of samples compared to low scores (<45) in 24.4% (9/46) corresponding to values found in the literature for response to neoadjuvant bladder cancer therapy (23).

Although level I evidence has shown the survival benefit of neoadjuvant chemotherapy (NAC) in MIBC (15), it has not been widely endorsed due to concerns about overtreatment, the lack of efficiency among caregivers, and concern about ineffective therapy that may lead to surgical and disease progression (24). Indeed, complete pathologic response can be observed in 20–40% of patients who receive NAC while 30% show no response or progression (25). Applying EVOC to neoadjuvant bladder cancer treatment could potentially improve the percentage of responding patients and allow those who are not expected to respond to proceed directly to cystectomy.

The potential advantage of a predictive platform may be even greater in other cancer types. The reported response of pancreatic cancer to FOLFIRNOX (folinic acid, fluorouracil, irinotecan hydrochloride, and oxaliplatin) is less than 50% (26) and the response of sarcomas to a variety of chemotherapies can be as low as 20% (27). Applying the EVOC platform to cancers that show low response rates may significantly improve clinical outcomes since patients who progress on treatment often do not have an additional opportunity for therapy due to their advance diseased state.

Most of the samples analyzed were from bladder cancer (*n* = 16) and pancreatic cancer (*n* = 9), demonstrating the predictive accuracy of the platform in a variety of different solid tumor types and origins. It was necessary to recruit from a basket of different cancer types and treatment stages due to the significant challenge of assembling a large cohort of patients who were able to undergo a research biopsy prior to treatment, who received the intended to treat drug and who underwent imaging within 3 months for RECIST. Eleven different cancer treatments (including combinations of multiple drugs) were evaluated across all 34 cancer samples, demonstrating the robustness of the platform in testing a wide spectrum of therapies. As nearly all samples tested were evaluated with chemotherapy, further investigation of the accuracy of the system in evaluating response to biological therapies and small molecules is needed. An earlier report about the EVOC platform showed clear responses and detailed analysis using small molecule pathway inhibitors suggesting that the predictive accuracy of the platform may potentially be preserved across other therapeutic modalities (11).

The utility of the functional EVOC platform may increase when combined with genomic sequencing which can suggest potentially efficacious drugs that can then be further evaluated in the functional assay. Ideally, rapid genomic sequencing (less than 24 h) would be performed prior to preparing the EVOC to establish an initial selection of drugs that are associated with specific genomic mutations. One approach to accomplishing such a goal would be to use a rapid amplicon-based library preparation protocol (2.5 h), as opposed to other amplicon or hybrid capture approaches (which may require 8–48 h). The use of a mutation panel focused specifically on genes with available therapies (~60 somatic gene mutations at 2000× depth), could allow for highly focused genomic-functional therapy selection.

This first clinical study of an EVOC precision medicine platform cultured for more than 5 days showed that this technology has high sensitivity and specificity. The main limitation of the study is the relatively small cohort of patients with different types of cancers. Larger studies are needed to confirm these results and strengthen the overall statistical outcomes. Additionally, the clinical utility of the platform should be validated in interventional settings where EVOC would be used to select the appropriate therapy for patient treatment. Such studies should use longer-term oncological outcomes such as progression-free survival and overall survival to determine whether EVOC-based personalized medicine confers clinical advantages for patients.

## Data availability statement

The datasets presented in this article are not readily available due to privacy and ethical restrictions. Requests to access the datasets should be directed to shaygo1@gmail.com.

## Ethics statement

The studies involving humans were approved by Rabin Medical Center Ethics Committee; Kaplan Medical Center Ethics Committee; Shaare Zedek Medical Center Ethics Committee; Hadassah University Medical Center Ethics Committee; Sheba Medical Center Ethics Committee; Tel Aviv Sourasky Medical Center Ethics Committee. The studies were conducted in accordance with the local legislation and institutional requirements. The participants provided their written informed consent to participate in this study.

## Author contributions

SG, SS, RS, ER, and VB: conceptualized the study and designed it obtained resources, collected and analyzed the data, and wrote the manuscript draft and revised it. GN, GC, DaK, SA, LT, AdZ, HamS, HagS, GM, ES, NG, ZD, RB, CS, TG, SH, DL, SB, YR, LA, AH, HN, TP, AvZ, BC, DmK, and AS collected and analyzed the data and revised the manuscript draft. All authors contributed to the article and approved the submitted version.

## Abbreviations

CR: complete response
CT: computed tomography
DMEM: Dulbecco’s modified eagle’s medium
EVOC: *ex vivo* organ culture
FCS: fetal calf serum
FFPE: formalin-fixed paraffin embedding
H&E: hematoxylin and eosin
MIBC: muscle-invasive bladder cancer
NGS: next generation sequencing
NPV: negative predictive value
PD: progressive disease
PDX: patient-derived xenograft
PET: positron-emission tomography
PFA: paraformaldehyde
PPV: positive predictive value
RA: radical cystectomy
RECIST: Response evaluation criteria in solid tumors
SD: stable disease
TURBT: transurethral resection of bladder tumor
