# Supplementary material for: A clinical evaluation of an ex vivo organ culture system to predict patient response to cancer therapy
Source: Front Med (Lausanne). 2023 Sep 28;10:1221484. doi: 10.3389/fmed.2023.1221484 (PMC10569691; doi:10.3389/fmed.2023.1221484)

**Supplementary Table 2. Study sites and IRB approval numbers**

Locations and ethics approval numbers of all sites participating in the clinical trial.


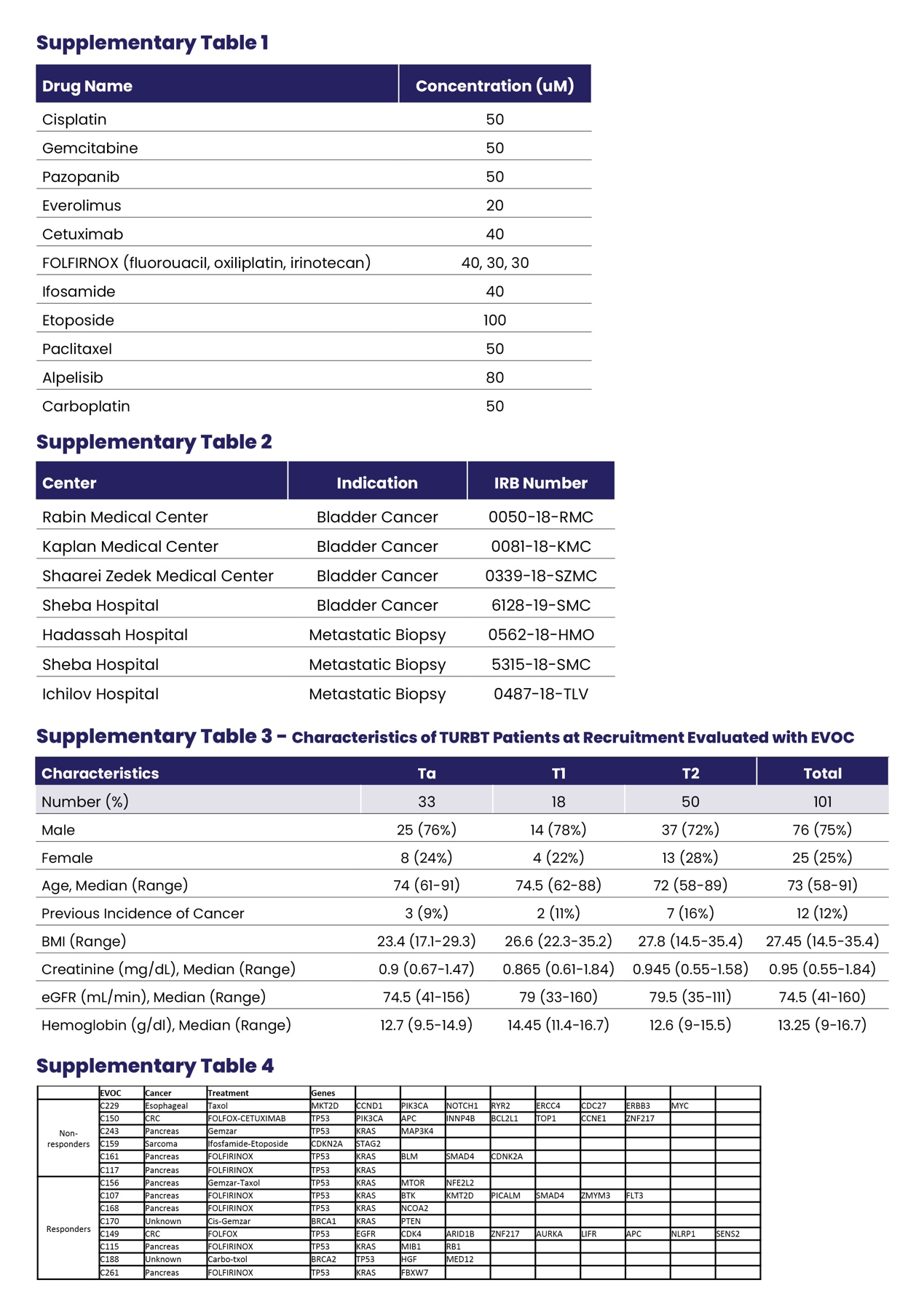

Supplement: Supplementary file 2 [file Table_2.docx]
