# Supplementary material for: A clinical evaluation of an ex vivo organ culture system to predict patient response to cancer therapy
Source: Front Med (Lausanne). 2023 Sep 28;10:1221484. doi: 10.3389/fmed.2023.1221484 (PMC10569691; doi:10.3389/fmed.2023.1221484)
Supplement: Supplementary file 3 [file Table_3.docx]

**Supplementary Table 3.** **Baseline clinical characteristics of patients who underwent transurethral resection of bladder tumor and had a viable cancer sample as assessed by the laboratory**

Below is a table of the age, sex and clinical characteristics of all patients who’s samples were evaluated in the EVOC system, irrespective of cancer stage.

**
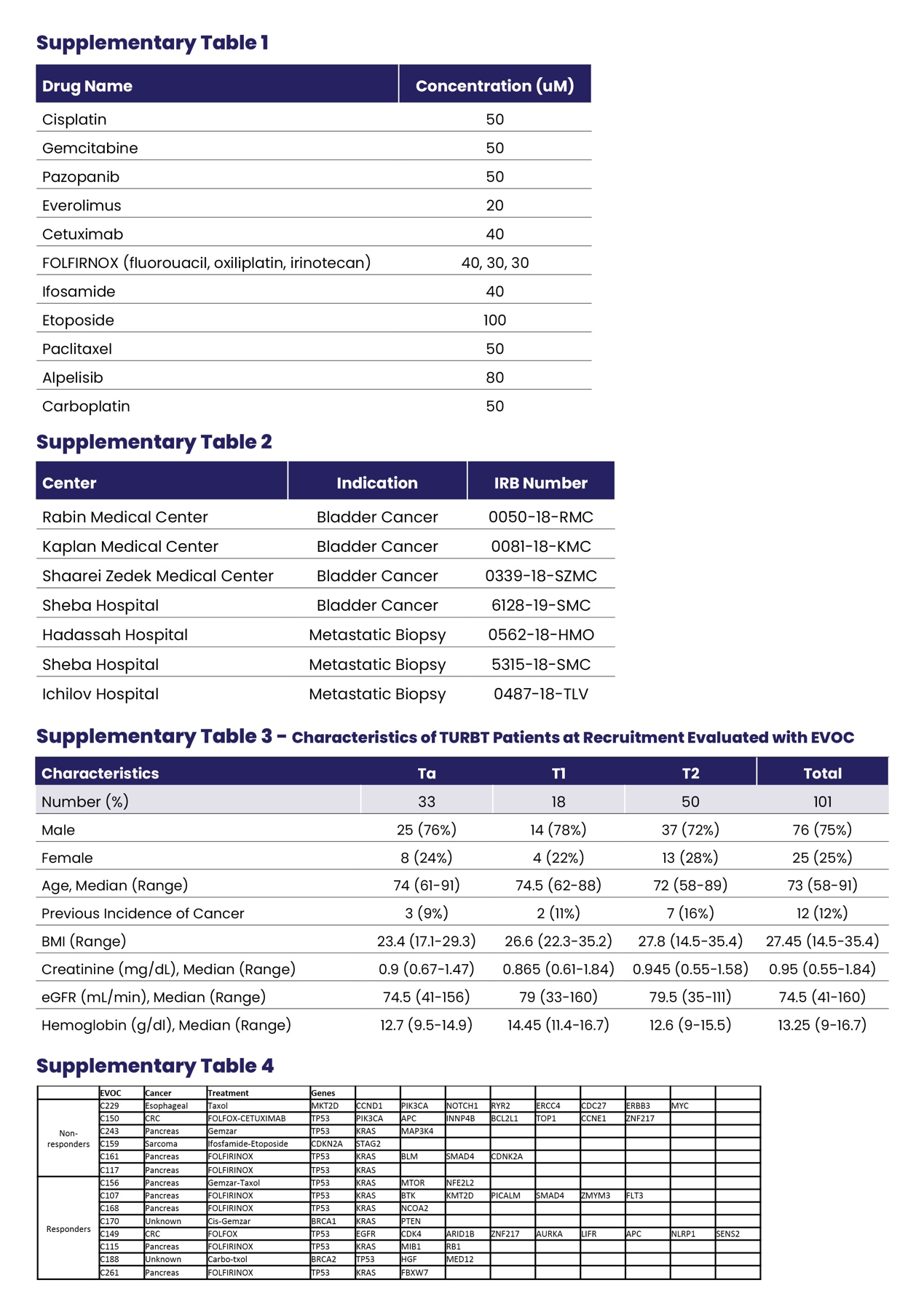
**
