# Supplementary material for: A clinical evaluation of an ex vivo organ culture system to predict patient response to cancer therapy
Source: Front Med (Lausanne). 2023 Sep 28;10:1221484. doi: 10.3389/fmed.2023.1221484 (PMC10569691; doi:10.3389/fmed.2023.1221484)
Supplement: Supplementary file 4 [file Table_4.docx]

**Supplementary Table 4. Somatic mutations found in patients who had solid tumors with metastases**

Fifty-seven somatic mutations found in patients who had solid tumors with metastases were analyzed.

|  | **EVOC** | **Cancer** | **Treatment** | **Genes** |  |  |  |  |  |  |  |  |  |
| --- | --- | --- | --- | --- | --- | --- | --- | --- | --- | --- | --- | --- | --- |
| Non-responders | C229 | Esophageal | Taxol | MKT2D | CCND1 | PIK3CA | NOTCH1 | RYR2 | ERCC4 | CDC27 | ERBB3 | MYC |  |
|  | C150 | Colorectal cancer | FOLFOX-bevacizumab | TP53 | PIK3CA | APC | INNP4B | BCL2L1 | TOP1 | CCNE1 | ZNF217 |  |  |
|  | C243 | Pancreas | Gemcitabine | TP53 | KRAS | MAP3K4 |  |  |  |  |  |  |  |
|  | C159 | Sarcoma | Ifosfamide-Etoposide | CDKN2A | STAG2 |  |  |  |  |  |  |  |  |
|  | C161 | Pancreas | FOLFIRINOX | TP53 | KRAS | BLM | SMAD4 | CDNK2A |  |  |  |  |  |
|  | C117 | Pancreas | FOLFIRINOX | TP53 | KRAS |  |  |  |  |  |  |  |  |
| Responders | C156 | Pancreas | Gemcitabine-paclitaxel | TP53 | KRAS | MTOR | NFE2L2 |  |  |  |  |  |  |
|  | C107 | Pancreas | FOLFIRINOX | TP53 | KRAS | BTK | KMT2D | PICALM | SMAD4 | ZMYM3 | FLT3 |  |  |
|  | C168 | Pancreas | FOLFIRINOX | TP53 | KRAS | NCOA2 |  |  |  |  |  |  |  |
|  | C170 | Unknown | cisplatin-gemcitabine | BRCA1 | KRAS | PTEN |  |  |  |  |  |  |  |
|  | C149 | Colorectal cancer | FOLFOX | TP53 | EGFR | CDK4 | ARID1B | ZNF217 | AURKA | LIFR | APC | NLRP1 | SENS2 |
|  | C115 | Pancreas | FOLFIRINOX | TP53 | KRAS | MIB1 | RB1 |  |  |  |  |  |  |
|  | C188 | Unknown | Paclitaxel and carboplatin | BRCA2 | TP53 | HGF | MED12 |  |  |  |  |  |  |
|  | C261 | Pancreas | FOLFIRINOX | TP53 | KRAS | FBXW7 |  |  |  |  |  |  |  |

FOLFIRINOX = leucovorin calcium (folinic acid), fluorouracil, irinotecan hydrochloride, oxaliplatin; FOLFOX = leucovorin calcium (folinic acid), fluorouracil, oxaliplatin
