# Supplementary material for: A clinical evaluation of an ex vivo organ culture system to predict patient response to cancer therapy
Source: Front Med (Lausanne). 2023 Sep 28;10:1221484. doi: 10.3389/fmed.2023.1221484 (PMC10569691; doi:10.3389/fmed.2023.1221484)

**Supplementary Figure 1. CONSORT flow chart of patients with muscle-invasive bladder cancer (MIBC) patients who participated in the study.** 111 potential MIBC patients were enrolled in the study, yielding 50 patients with MIBC and 16 who completed treatment with EVOC scores.


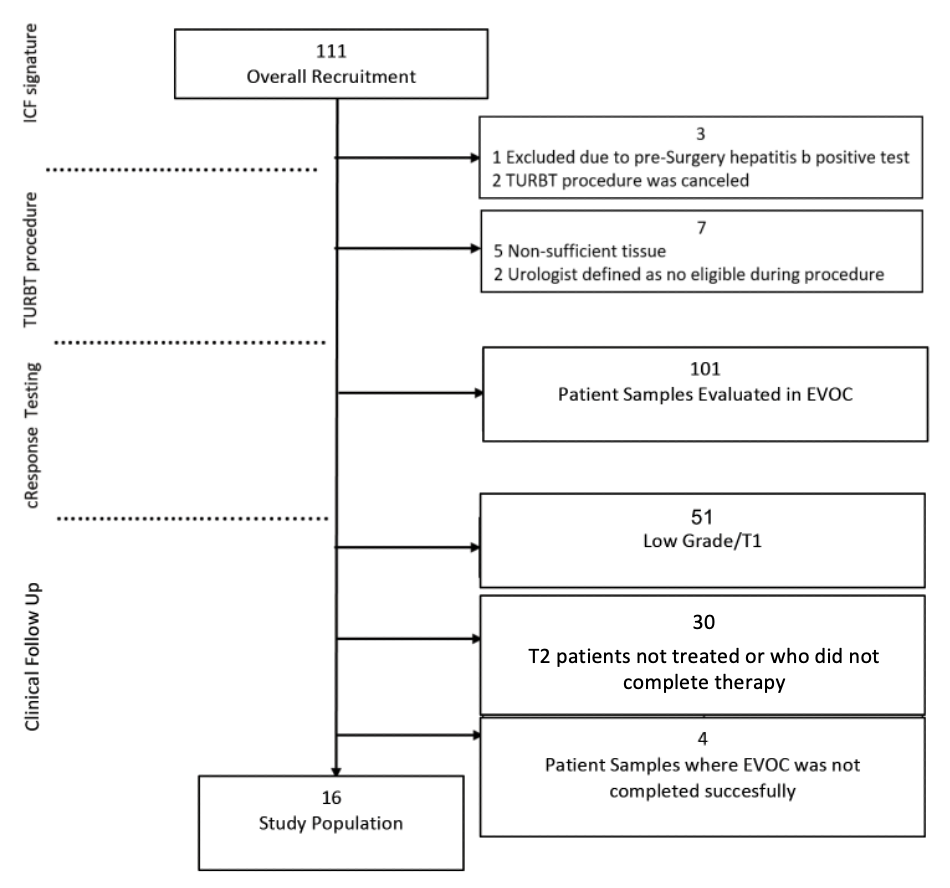

Supplement: Supplementary file 5 [file Data_Sheet_1.docx]
