# Supplementary material for: A clinical evaluation of an ex vivo organ culture system to predict patient response to cancer therapy
Source: Front Med (Lausanne). 2023 Sep 28;10:1221484. doi: 10.3389/fmed.2023.1221484 (PMC10569691; doi:10.3389/fmed.2023.1221484)

**Supplementary Figure 2. Urothelial carcinoma responder and non-responder.** Additional images of a patient that did not respond to neo-adjuvant therapy (C237, Score=14) and a patient that did respond to therapy (C315, Score=88).


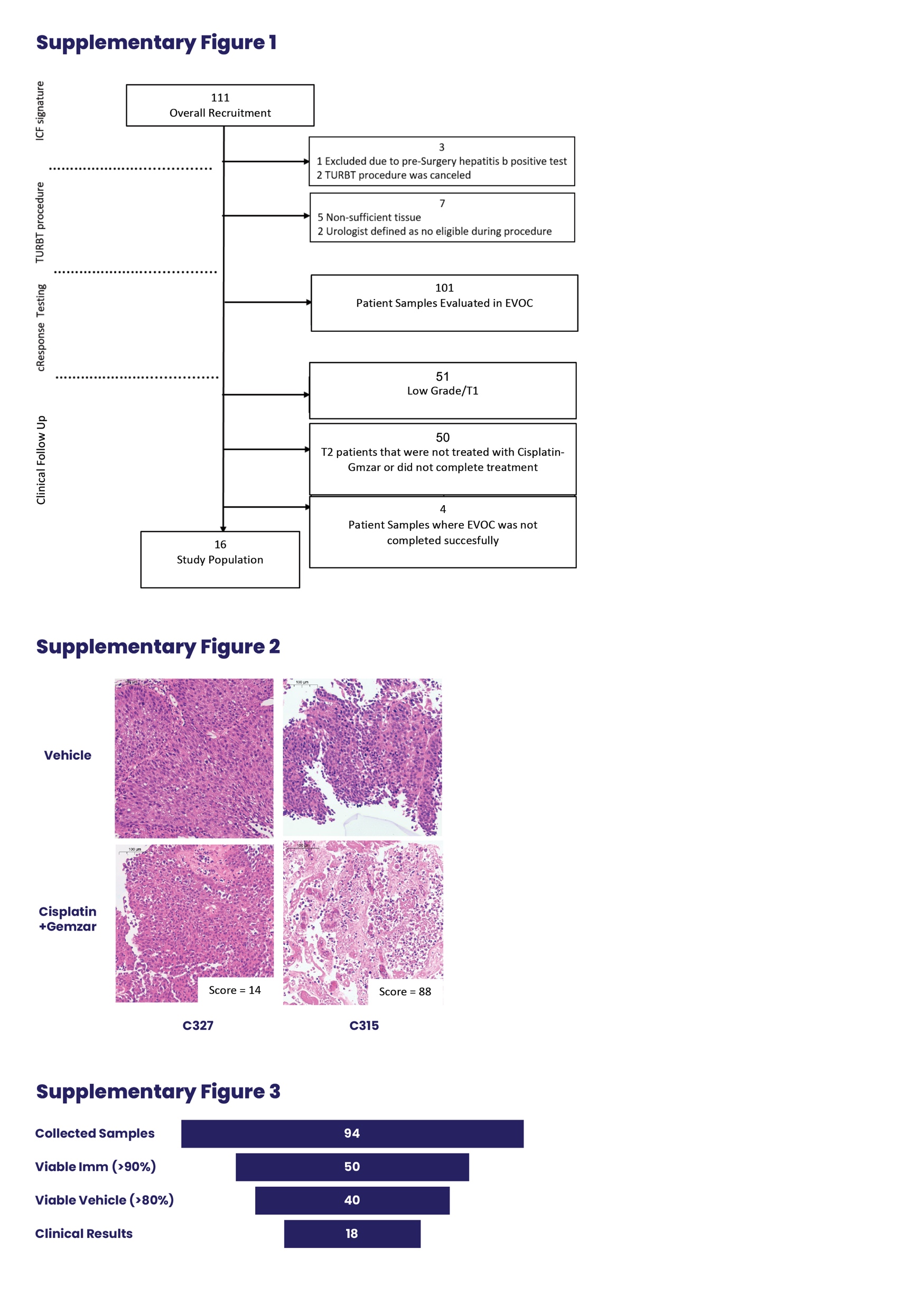

Supplement: Supplementary file 6 [file Data_Sheet_2.docx]
