# Supplementary material for: A clinical evaluation of an ex vivo organ culture system to predict patient response to cancer therapy
Source: Front Med (Lausanne). 2023 Sep 28;10:1221484. doi: 10.3389/fmed.2023.1221484 (PMC10569691; doi:10.3389/fmed.2023.1221484)

**Supplementary Figure 3. Patient distribution in the core needle biopsy study.** The number of patients biopsy samples collected, those with viable immediates (>90% live cancer), those with viable vehicles from EVOC (>80% live cancer) and those with clinical results are presented.


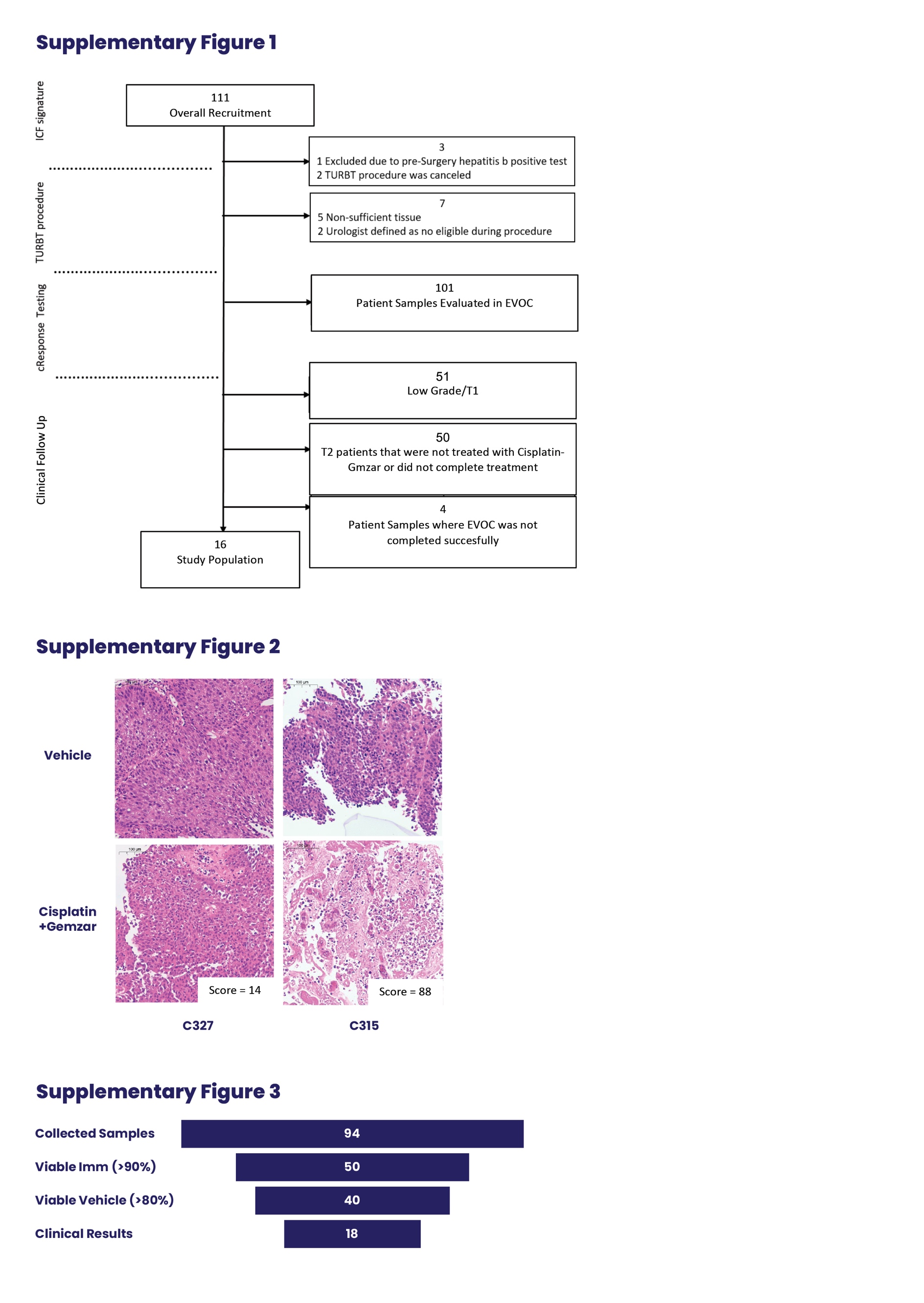

Supplement: Supplementary file 7 [file Data_Sheet_3.docx]
